# Thoracic Wounds—Experience at an Advanced Surgical Center

**Published:** 1918-11

**Authors:** 


**Thoracic Wounds—Experience at an Advanced Surgical Center.** A. Rees and Gerard S. Hughes, Lancet, Jan. 12, report on 140 chest wounds. Primary haemorrhage is the principal cause of death early. This is due mainly to bleeding from the intercostal artery but also to that from the pulmonary vessels. While the latter is arrested by pulmonary collapse, the tamponment of the vessels by the blood collected in the pleural cavity cannot be efficacious till a very abundant, even fatal haemorrhage has occurred. In widely open wounds with trauma of the lung, 27 deaths occurred in 44 cases—61%. If the wound is not closed in 48 hours, infection is inevitable. Simple lavage is often sufficient to close a small open wound. Tamponing is insufficient in large wounds and dangerous on account of liability to sepsis. Suture of the wound, with or without extraction of the projectile is to be recommended in all cases of open wounds for infection of the pleura can be avoided in half the cases. The operation is done after shock has passed, under light general anaesthesia with chloroform and warm oxygen. Extraction of projectiles from the pulmonary parenchyma and suture of the visceral pleura prolong the operation and should not be practiced as a rule. Closed wounds (haemothorax and pneumothorax closed): 72 cases, 14 deaths, 19.3%. The complication to be feared is infection, indicated by the persistance of tachycardia, fever and acute pain on percussion of the wounded side.

